# Tigecycline Resistant *Klebsiella pneumoniae* Isolated from Austrian River Water

**DOI:** 10.3390/ijerph14101169

**Published:** 2017-10-03

**Authors:** Alexander Hladicz, Clemens Kittinger, Gernot Zarfel

**Affiliations:** 1 Center for Molecular Biology, University of Vienna, 1030 Vienna, Austria; alexander.hladicz@gmx.at; 2 Institute of Hygiene, Microbiology and Environmental Medicine, Medical University of Graz, Neue Stiftingtalstrasse 2, 8010 Graz, Austria; clemens.kittinger@medunigraz.at

**Keywords:** ramA, efflux pump, multilocus sequence typing, surface water

## Abstract

Antibiotic-resistant bacteria are spreading worldwide in medical settings but also in the environment. These resistant bacteria illustrate a major health problem in our times, and last-line antibiotics such as tigecycline represent an ultimate therapy option. Reports on tigecycline non-susceptible *Enterobacteriaceae* are presented with regard to medical settings but are rare with that for the environment. The aim of this study was to characterize two tigecycline non-susceptible *Klebsiella pneumoniae* isolates from the river Mur, and to question the resistance mechanism. The screening for chromosomal mutations revealed a deletion and a silent point mutation in one isolate and a point mutation in the other isolate all within the *ramR* allele. RamR acts as repressor and prevents overexpression of *ramA*. These mutations are likely to cause a resistant phenotype due to the overexpression of AcrAB-TolC. MLST revealed that the isolates belonged to two unrelated MLST types (ST2392 and ST2394). Both isolates only revealed resistance to tigecycline and tetracycline. This is one of the rare reports of tigecycline-resistant *Klebsiella pneumoniae* from surface water. The presence of two genetically different isolates suggests that the river water may bear substances that favor mutations that can lead to this efflux pump-driven resistance.

## 1. Introduction

The emergence of antibiotic resistances is a worldwide rising phenomenon. It is not restricted to clinical settings and it reaches environmental settings and their associated ecological habitats. In particular, surface waters such as rivers, lakes or coastal waters act as reservoirs for resistant bacteria owing to anthropogenic activities and influences such as industrial or urban sewage [1,2,3,4,5]. The discharge of resistant bacteria in combination with antibiotics and/or other chemical compounds into the water bodies is likely to select for antibiotic resistances within microbial communities [6,7,8]. Therefore, effluents or insufficient water management promotes the distribution of resistant bacteria and facilitates the spread of resistance genes [9]. This trend of emerging antibiotic-resistant bacteria speeds up by the overuse of antibiotics in human and veterinary medicine, and a subsequent release of these substances into the environment [10].

The massive health problem that arises from the current situation concerns (opportunistic) pathogens that gained multidrug resistance (MDR) to a broad spectrum of antibiotics. ESBL-(extended-spectrum b-lactamase) or carbapenemase-producing *Enterobacteriaceae*, notably *Klebsiella pneumoniae*, are described not only in clinical but also in different aquatic settings all around the world, including Austria [2,3,4,11,12,13].

Last-resort antibiotics act as ultimate force to overcome those multiresistant strains. Tigecycline is such an antibiotic and is often the last or the penultimate choice (besides colistin) to treat infections caused by those pathogens [14,15]. Hence, occurrence of tigecycline resistance is a major threat to every medical institution. Cases of tigecycline non-susceptible *Klebsiella* *pneumoniae* in clinical settings are reported worldwide [16,17] but are rather rare regarding environmental settings.

There are different mechanisms that can lead to an acquired tigecycline resistance, most of them based on chromosomal mutations. Gene network of the efflux pump, AcrAB-TolC is associated with tigecycline non-susceptibility and its regulators has been analyzed with regard to tigecycline non-susceptibility in prior studies. In particular, mutation in the repressors RamR, MarR and SoxR of the regulators (RamA, MarA and SoxS) of the efflux pump were found to be responsible [18,19,20,21,22].

An additional mutation in the ribosomal RPS10 protein, which is located close to the ribosomal binding site of tigecycline is likely to influence the binding properties between the ribosome and tigecycline [21].

The aim of this study was to elucidate the resistance mechanism that causes tigecycline non-susceptibility and to question whether this mechanism is plasmid or chromosomally mediated. In order to detect a potential plasmid-encoded resistance mechanism, transformation experiments were performed.

## 2. Material and Methods

### 2.1. Sample Collection

Water samples were taken for microbiological investigations during a survey from the river Mur in the center of Graz (47°4′38″ N; 15°25′60″ E); each sample in two sterile 500 mL glass flasks, 30 cm below the river surface, 50 cm apart from the river bank.

### 2.2. Isolation of Bacteria

Samples were filtered using Microfil^®^ S device (Merck, Vienna, Austria) with 0.45 μm pore filters in 4 times 250 mL portions. For each sampling, two filters were put on chromID^™^ ESBL Agar (bioMérieux Austria GmbH, Vienna, Austria) and two on chromID^™^ CARBA Agar (bioMérieux). ChromID^™^ agars were incubated for 24 h at 37 °C. Colonies were assessed and picked according to the manufacturer’s manual. For pure cultures, colonies were transferred to blood agar and Endo agar (24 h, 37 °C) and species were finally identified with MALDI-TOF, (Vitek^®^ MS, bioMérieux Austria GmbH, Vienna, Austria).

Thereby *Klebsiella pneumoniae* isolates could be recovered on chromID^™^ CARBA Agar (MurTR-KL001 on 2 February 2016; and MurTR-KL002 on 11 February 2016).

### 2.3. Antimicrobial Susceptibility Testing

Susceptibility testing was performed according to the Clinical and Laboratory Standards Institute (CLSI) guidelines or as recommended by the European Committee on Antimicrobial Susceptibility testing (EUCAST) using BD BBLTM, Sensi-Disc^™^ paper discs (Becton, Dickinson and Company, Sparks, MD, USA) [23,24].

The inhibition zone diameters were interpreted according to EUCAST guidelines with the exception for tetracycline, chloramphenicol and nalidixic acid, which were evaluated in conformity with the Clinical Laboratory Standards Institute (CLSI) guidelines. EUCAST guidelines were chosen as they are the clinical standard for Europe; whenever EUCAST criteria were not available CLSI standards were used.

The following antibiotics were used: amoxicillin/clavulanic acid (20 μg/10 μg), piperacillin/tazobactam (100 μg/10 μg), cefalexin (30 μg), cefuroxime (30 μg), cefoxitin (30 μg), cefotaxime (5 μg), ceftazidime (10 μg), cefepime (30 μg), imipenem (10 μg), meropenem (10 μg), amikacine (30 μg), gentamicin (10 μg), trimethoprim/sulfamethoxazole (1.25 μg/23.75 μg), ciprofloxacin (5 μg), moxifloxacin (5 μg), tetracycline (30 μg), nalidixic acid (30 μg) chloramphenicol (30 μg).

To determine tigecycline and colistin susceptibility, Etests^®^ (bioMérieux Austria GmbH, Vienna, Austria) according to EUCAST guidelines for tigecycline and colistin were performed as described previously [25,26].

*Escherichia coli* ATCC 25922 and *Pseudomonas aeruginosa* ATCC 27853 were used as control strains in all conducted tests. 

### 2.4. Plasmid Replicon Typing

Identification of replicon types of the 18 major plasmid incompatibility (Inc) groups present in *Enterobacteriaceae* was performed by multiplex PCR. 

Standard PCR protocols and conditions were used in the following way: initial denaturation at 94 °C for 5 min; 30 cycles of 94 °C for 1 min, 60 °C for 30 s and 72 °C for 1 min; and a final incubation for 5 min at 72 °C. We used Taq DNA polymerase and dNTPs from QIAGEN (Hilden, Germany), and a T3000 Biometra thermocycler (Biometra, Gottingen, Germany).

The protocol allows detection of the following Inc groups: Hl1, Hl2, I1-Iγ, X, L/M, N, FIA, FIB, W, Y, P, FIC, A/C, T, FII_s_, F, K, B/O [27].

### 2.5. Transformation by Electroporation

Preparation of Plasmid-DNA was performed with the QIAprep Spin Miniprep Kit (250) (QIAGEN).

Plasmid-DNA was desalted before electroporation, and therefore 2–3 µL of plasmid-DNA were transferred on a MF^TM^ Membrane Filter (0.025 µm VSWP, Merck), which was placed on the surface of double distilled water. Dialysis was performed for about 15 min.

Competent cells were made with two overnight cultures (each 50 mL, OD of 0.4), which were incubated on ice for 25 min, therefore reaction tubes were cooled in advance, followed by centrifugation for 10 min at 4 °C and 4.000 rpm (Eppendorf, Centrifuge 5810R). After decantation, pellets were re-suspended in 100 mL ice-cold glycerine solution (10%). After repeating this step, an additional washing step was performed, and the two pellets together were re-suspended in 10 mL glycerine solution (10%). A last washing step and resuspension were performed with 1 mL glycerine solution (10%). Aliquots of 50 µL were prepared and stored at −20 °C.

Electroporation was performed with 2 µL Plasmid-DNA and 40 µL of competent cells. Reaction tubes were cooled in advance and the DNA-cell suspension was incubated on ice for 5 min. Subsequently, the cell suspension was transferred into a sterile electro-cuvette, and transformation was performed at 2500 V using the electroporator (Eppendorf Eporator^®^). After the transformation, 400 µL of fresh LB liquid media were added and the cell suspension was re-transferred into the reaction tube. Incubation was performed for 40 min at 37 °C. Afterwards 100 µL of the cell suspension were plated on selection LB (lysogeny broth) plates (tetracycline 3 µg/mL or tigecycline 1 µg/mL) and a final incubation was performed over night at 37 °C.

### 2.6. Multilocus Sequence Typing (MLST)

MLST was performed for *Klebsiella pneumoniae* according to the Institute Pasteur MLST (http://bigsdb.web.pasteur.fr/klebsiella/klebsiella.html).

### 2.7. Screening for Mutations

The genes *ramR*, *marR*, *soxR* and *rpsJ* were amplified and sequenced with the primers described previously [18,21].

Standard PCR protocols and conditions were used in the following way: initial denaturation at 94 °C for 5 min; 35 cycles of 94 °C for 30 s, 52 °C for 1 min and 72 °C for 1 min; and a final incubation for 5 min at 72 °C. We used Taq DNA polymerase and dNTPs from QIAGEN (Hilden, Germany), and a T3000 Biometra thermocycler (Biometra, Germany).

Sequencing was performed with the Mix2Seq Kit (Eurofins Genomics).

Sequence analysis was performed with Serial Cloner v2.6 and BLAST (Basic Local Alignment Search Tool, https://blast.ncbi.nlm.nih.gov/Blast.cgi).

## 3. Results

Two *Klebsiella pneumoniae* isolates (MurTR-KL001 and MurTR-KL002) were randomly sampled from the river Mur during a study not linked to tigecycline.

### 3.1. Antimicrobial Susceptibility Testing

Both isolates revealed only resistance to tetracycline and tigecycline but stayed susceptible to all other tested antibiotics. Isolate MurTR-KL001 revealed a minimal inhibition concentration to tigecycline of 4 µg/mL and MurTR-KL002 of 8 µg/mL (Table 1).

### 3.2. Genetic Analyses

The two isolates belonged to two different unrelated MLST types: ST2392 (rpoB:1, gapA:2, mdh:172, pgi:1, phoE:9, infB:1, tonB:116) and ST2394 (rpoB:4, gapA:126, mdh:1, pgi:1, phoE:4, infB:3, tonB:351). Both MLST profiles had not been described prior to our study. Notably, *tonB* of MurTR-KL002 revealed a new allele (*tonB* 351) (Table 1).

### 3.3. Determination of a Plasmid-Encoded Resistance Mechanism

The plasmid type FII_S_ could be determined in both isolates. Transformation experiments revealed that no resistance was transferred by plasmids.

### 3.4. Determination of a Chromosomally-Encoded Resistance Mechanism

All alleles of *soxR*, *marR* and *rpsJ* were identical with sequences from tigecycline susceptible *Klebsiella pneumoniae* strains previously described (GenBank accession numbers: CP000647.1 [22], CP009461.1 [28], CP003999.1 [29], KC843636.1 [21]). Even though *marR* of MurTR-KL002 harbored a silent mutation (C270A), no other mutations within these genes could be observed.

With regard to the reference strain *Klebsiella pneumoniae* subsp. *pneumoniae* MGH 78578 (GenBank accession number: CP000647.1 [22]), mutations in both isolates could be observed within the *ramR* allele. *RamR* of MurTR-KL001 primarily harbored a four base pair deletion (∆ 518–521CCCG) resulting in a frameshift. Secondarily, a silent point mutation was on position 291 with a G to A mutation. *RamR* of MurTR-KL002 harbored a point mutation (152A > C), which resulted in an amino acid substitution (K51T) (Table 1).

## 4. Discussion

Tigecycline non-susceptible *Klebsiella pneumoniae* were recently isolated from heavily polluted coastal waters in Brazil [30] and less recently from hospital sewage in Saudi Arabia [31]. Other resistant *Enterobacteriaceae* could be recovered from drinking water samples in India [32].

However, such reports from more “decent” aquatic settings are rare in the current literature. Recent cases of tigecycline-resistant *Klebsiella pneumoniae* were reported from urban surface waters in Brazil: from a river downstream of a wastewater treatment plant, in Curitiba [1], and from an urban lake and reservoir in the city of Sao Paulo [4].

Even though the two isolates belong to two newly described and distinctly different MLST types, they seem to share the same resistance mechanism (even though owing two different mutations), which could indicate a common selective pressure. They were susceptible to all other tested antibiotics. Taking also into account that both isolates belong to two new MLST types it is very unlikely that these isolates are a contamination from a clinical source. Although low concentrations of antibiotics can cause an ecological shift towards less susceptible bacteria, it is rather unlikely that the river Mur was contaminated with tigecycline [33].

A recent study demonstrated cross-resistance to antibiotics, including tetracycline, in association with the resistance to linalool, a component of basil oil that is used as a natural preservative. The increased resistance to linalool was accompanied by the overexpression of the AcrAB efflux pump suggesting linalool as potential substrate [34]. A similar cross-resistance to antibiotics was observed in association with the resistance to pine oil and the tolerance to solvents; in both cases resistance correlated with the activity of the AcrAB efflux pump [35]. Decreased susceptibility to triclosan, a biocide, was also reported in the course of *acrAB* overexpression. Moreover, the AcrAB efflux pump extrudes dyes and detergents, and appears to play a more crucial role, as it is embedded in fundamentally physiological functions; for instance, in cell-to-cell communication and in virulence. It appears plausible that a cross-resistance to an antibiotic could easily fall within a more fundamentally microbial purpose as long as the overexpression of the efflux pump is favored within an ecological and physiological setting. In that manner, higher concentrations of any potential substrate could select for, i.e., a tigecycline resistance [36,37,38].

Nikaido et al. and Baucheron et al. proposed a mechanism of induction for the AcrAB locus. They suggested that indole and bile bind to RamR, thereby inhibiting its repressing effect on *ramA* transcription, and therefore promoting the induction of the *ramR* and *acrAB* locus. Yamasaki et al. further reported that different substrates can bind to RamR due to a flexible binding pocket and upon binding the DNA binding affinity of RamR decreases. Therefore, substrates could act as extracellular signals that force subsequent induction of *ramA* and *acrAB* expression, whenever the efflux system is overloaded. However, a mutation within RamR can also lead to the induction of the efflux pump resembling a permanent sensing signal. That arrangement may endure in a suitable ecological or physiological condition [39,40].

RamR represents a genetic hotspot for mutations as far as clinical Klebsiella pneumoniae isolates are concerned [18,19,20,21]. None of the reported mutations are identical, the closest mutation to the MurTR-KL001 isolate was described by Rosenblum et al. [41]. Nevertheless, reports of aquatic isolates harboring such mutations are absent in the current literature. 

## 5. Conclusions

The presence of two genetically different isolates suggests that river water may bear substances that favor mutations that can lead to this efflux pump-driven resistance. The origin of these substances (e.g., triclosan or heavy metals) may be waste water or surface run-off after rainfall. Therefore, the occurrence and impact on human health of such mutations in bacteria in surface waters must be further investigated. 

## Figures and Tables

**Table 1:** Characterization of tigecycline-resistant *Klebsiella pneumoniae* isolates.

Isolate	MLST	*ramR* Mutation	MIC Tetracycline	MIC Tigecycline
MurTR-KL001	ST2392	291G > A (V97V); ∆ 518–521,	8 µg/mL	4 µg/mL
MurTR-KL002	ST2394	152A > C (K51T).	8 µg/mL	8 µg/mL
